# Performance of Two Risk-Stratification Models in Hospitalized Patients With Coronavirus Disease

**DOI:** 10.3389/fmed.2020.00518

**Published:** 2020-08-14

**Authors:** Rong Xu, Keke Hou, Kun Zhang, Huayan Xu, Na Zhang, Hang Fu, Linjun Xie, Ran Sun, Lingyi Wen, Hui Liu, Zhigang Yang, Ming Yang, Yingkun Guo

**Affiliations:** ^1^Key Laboratory of Birth Defects and Related Diseases of Women and Children of Ministry of Education, Department of Radiology, West China Second University Hospital, Sichuan University, Chengdu, China; ^2^Department of Radiology, Public Health Clinical Center of Chengdu, Chengdu, China; ^3^Department of Radiology, West China Hospital, Sichuan University, Chengdu, China; ^4^Department of Respiratory Medicine, Public Health Clinical Center of Chengdu, Chengdu, China

**Keywords:** risk-stratification, coronavirus disease, MuLBSTA score, CURB65 score, ICU

## Abstract

**Background:** Despite an increase in the familiarity of the medical community with the epidemiological and clinical characteristics of coronavirus disease 2019 (COVID-19), there is presently a lack of rapid and effective risk stratification indicators to predict the poor clinical outcomes of COVID-19 especially in severe patients.

**Methods:** In this retrospective single-center study, we included 117 cases confirmed with COVID-19. The clinical, laboratory, and imaging features were collected and analyzed during admission. The Multi-lobular infiltration, hypo-Lymphocytosis, Bacterial coinfection, Smoking history, hyper-Tension and Age (MuLBSTA) Score and Confusion, Urea, Respiratory rate, Blood pressure, Age 65 (CURB65) score were used to assess the death and intensive care unit (ICU) risks in all patients.

**Results:** Among of all 117 hospitalized patients, 21 (17.9%) patients were admitted to the ICU care, and 5 (4.3%) patients were died. The median hospital stay was 12 (10–15) days. There were 18 patients with MuLBSTA score ≥ 12 points and were all of severe type. In severe type, ICU care and death patients, the proportion with MuLBSTA ≥ 12 points were greater than that of CURB65 score ≥ 3 points (severe type patients, 50 vs. 27.8%; ICU care, 61.9 vs. 19.0%; death, 100 vs. 40%). For the MuLBSTA score, the ROC curve showed good efficiency of diagnosis death (area under the curve [AUC], 0.956; cutoff value, 12; specificity, 89.5%; sensitivity, 100%) and ICU care (AUC, 0.875; cutoff value, 11; specificity, 91.7%; sensitivity, 71.4%). The K–M survival analysis showed that patients with MuLBSTA score ≥ 12 had higher risk of ICU (log-rank, *P* = 0.001) and high risk of death (log-rank, *P* = 0.000).

**Conclusions:** The MuLBSTA score is valuable for risk stratification and could effectively screen high-risk patients at admission. The higher score at admission have higher risk of ICU care and death in patients infected with COVID.

## Introduction

In December 2019, a series of pneumonia cases of unknown cause emerged in Wuhan, Hubei Province, China. Subsequently, a novel coronavirus was isolated and is known as the 2019 novel coronavirus (SARS-COV-2), which was designated as coronavirus disease 2019 (COVID-19) (1). With the worldwide prevalence and outbreak of COVID-19, the pressure regarding the prevention and treatment of this epidemic has intensified, and several local medical resources were seriously insufficient (2). Thus, understanding the risk stratification could help in the better allocation of the available medical resources as well as ensure appropriate clinical management of high-risk patients to improve the survival rate.

The clinical spectrum of COVID-19 infection appears to be wide, encompassing asymptomatic infection, mild upper respiratory tract illness, severe viral pneumonia with respiratory failure, shock, and even death. The current reported death rate is about 0.66–7.2% (1, 3–5). Some studies have published the risk factors that may be associated with poor prognosis, such as age or severe immune response (4–7). However, only a few studies focusing on clinical risk stratification, and the risk factors for in-hospital death or intensive care unit (ICU) care of patients were undefined.

An effective and comprehensive model for screening high-risk patients at admission is necessary for patients infected with SARS-COV-2. Therefore, we aimed to verify the efficacy of the Multi-lobular infiltration, hypo-Lymphocytosis, Bacterial coinfection, Smoking history, hyper-Tension, and Age (MuLBSTA) scale for mortality or ICU risk stratification in patients with COVID-19 and clarify the predictive value of the scale for poor prognosis.

## Methods

### Study Design and Data Collection

We recruited patients from January 1 to March 25, 2020 in this retrospective study. All patients were diagnosed with COVID-19 pneumonia according to RT-PCR. All laboratory and imaging reports during the hospitalization were recorded. The institutional ethic committee of our institutes approved this study (No. 2020.43).

History of exposure, clinical manifestations, laboratory findings, CT characteristics, and epidemiological and outcome data were obtained from the collection forms and electronic medical records from admission to discharge. All recorded data were independently reviewed by two researchers.

### CT Image Review

Signs and severity of lung lesions observed in Computed Tomography (CT) scans were evaluated, and lung involvement in each lobe was recorded. More than three lung lobes involvement were regarded as multi-lobular infiltrates. The “total severity score” was calculated by summing the five lobe scores (range: 0–25 points), and each of the five lung lobes were visually scored from 0 to 5 (8). All CT images were independently reviewed by two fellowship-trained cardiothoracic radiologists, and final decisions were reached by consensus.

### MuLBSTA Score and CURB 65 Score

The MuLBSTA Score were scaled in all patients. The score points as follows: Multi-lobular infiltrates (5 points), lymphocyte count ≤ 0.8 × 10^9^/L (4 points), bacterial coinfection (4 points, presented with bacteria positive by laboratory tests or sputum tests and there were consolidation signs on CT feature), acute smoker (3 points, and the patients who had quit-smoking history were scaled as 2 points), hypertension (2 points), and age ≥ 60 years (2 points). All patients received a total score calculation for MuLBSTA score. A score of 12 points was used as the cutoff value for mortality risk stratification [MuLBSTA 0-11 (low-risk” mortality) and MuLBSTA 12-22 (high-risk mortality)] (9).

Confusion, Urea, Respiratory rate, Blood pressure, Age 65 (CURB65) score were also scaled. The CURB65 is recommended for assessing the severity of pneumonia in hospital settings and the score system refer to previous studies (10, 11).

### Clinical Outcomes

Complications such as electrolyte disturbance, acute myocardial injury (AMI), acute kidney injury (AKI), acute respiratory distress syndrome (ARDS), and shock were recorded. The time from onset to admission and from admission to discharge were also recorded. Clinical outcomes included Death, ICU care, and recovery/discharge.

### Statistical Analysis

All tests were two-sided, and *P* < 0.05 was considered statistically significant. Categorical variables were described as frequency rates or percentages, and continuous variables were presented as mean (SD) or median (IQR). The mean values for continuous variables were compared using the independent *t*-tests when the data were normally distributed; otherwise, the Mann–Whitney test was used. For laboratory results, we also assessed whether the measurements were outside the normal range. The ROC curve was used to examine the efficacy of the MuLBSTA score for death or ICU. The Kaplan–Meier (K–M) survival analysis was performed to estimate the survival probabilities for COVID-19 infection by the log-rank test. All statistical analyses were performed using 22.2 SPSS software (Statistical Package for the Social Sciences).

## Results

### Presenting Characteristics

The study population included 117 hospitalized patients with confirmed cases of COVID-19. The youngest patient was 3 months old. Furthermore, 2 patients were admitted with mild symptoms and classified as common type, but then were classified as critically severe type after admission due to the symptoms rapidly aggravated. Moreover, there were 35 (29.9%) patients were older than 60 years. A total of 55 (47.0%) patients were men. The underlying diseases showed in Table 1. For the clinical severity type, 81 (69.2%) patients were common type, and 16 (13.7%) and 20 (17.1%) were severe and critically severe types, respectively. Among all patients, most of them (96, 82.1%) were discharged, 21 (17.9%) were admitted to the ICU, and 5 (4.3%) died.

**Table 1:** Baseline, clinical treatment, and outcome of all patients.

**Baseline**	**Total (*N* = 117)**	**Treatment**	**Total (*N* = 117)**
**Age (years)**		**Severity type**	
<40	37 (31.6%)	Common type	81 (69.2%)
40–59	45 (38.5%)	Severe type	16 (13.7%)
≥60	35 (29.9%)	Critically severe type	20(17.1%)
Male	55 (47.0%)	**Support Treatment**	
Hypertension	19 (16.2%)	High flow oxygen	19 (16.2%)
Diabetes	18 (15.4%)	Non-invasive Invasive	7 (6.0%) 6 (5.1%)
CVD	8 (6.8%)	CRRT	9 (7.7%)
CKD	5 (4.3%)	ECMO	1 (0.8%)
Obesity	16(13.8%)		
**Clinical outcome**			
ICU care	21 (17.9%)	**Medicine treatment**	
Discharged	96 (82.1%)	Antiviral	105 (89.7%)
Death	5 (4.3%)	Antibiotic	26 (22.2%)
**Interval time**		Thymalfasin	13 (11.1%)
Onset to admission (days)	5 (3–7)	Chinese medicinal	81 (69.2%)
Onset to discharge (days)	16 (14–23)	Interferon	104 (88.9%)
Admission to discharge (days)	12 (10–15)	Convalescent plasma	8 (6.8%)

*Date are n (%), mean ± SD, or median (IQR). CVD, cardiovascular disease; ICU, intensive care unit; CRRT, continuous renal replacement therapy; ECMO, extracorporeal membrane oxygenation*.

Of all 117 patients, the symptomatic treatment and invasive treatment were shown in Table 1. Among of nine patients with continuous renal replacement therapy, three were acute kidney injury, six were electrolyte disturbance or hypercytokinemia (2 patients had concurrent both of two conditions). And five patients were chronic kidney injury. One received extracorporeal membrane oxygenation due to the condition continues to deteriorate. Otherwise, the medicine treatment were also showed in Table 1, there were 8 (6.8%) patients repeatedly tested PCR positive, stayed in the hospital for more than 30 days, and received convalescent plasma (from cared patients).

### The Clinical Characteristics and CT Feature in Different Patients

In all patients, there was no statistical difference in body mass index (BMI) between common type and severe type patients. Among all severe type patients, the mean point of CURB65 score was 1.4 ± 1.5. And 12 (33.3%) of the patients underwent the prone position management.

The lymphocyte count and rate in severe type patient were significantly lower than those in common type patients (*P* < 0.05). Among the severe type patients, 21 (58.3%) and 28 (7.8%) presented with decreased lymphocyte count and rate, respectively, of which the percentages were higher than those in common type patients. Furthermore, the increased CPR level was higher in severe type patients than in common type patients (33 [91.7%] vs. 34 [42.0%]).

For the assessment of CT features, in all of 36 severe type patients, 34 (94.4%) and 35 (97.2%) showed more than 3 lung lobes affected and more than 2 mixture signs, respectively. The lung lobes involvement was shown in Table 2. Severe type patients had significantly higher lung severity scores than common type patients (7.8 ± 3.9 vs. 3.3 ± 2.4, *P* < 0.05).

**Table 2:** The clinical characteristics and CT feature in different type patients.

	**Total**	**Common type**	**Severe type**
	***N* = 117**	***N* = 81**	***N* = 36**
BMI	23.5 ± 3.8	23.4 ± 3.9	23.7 ± 3.9
CURB65 score	0.6 ± 1.0	0.2 ± 0.5	1.4 ± 1.2^*^
Prone positioning	12 (33.3%)	0 (0%)	12 (33.3%)
**Laboratory**			
Lymphocyte (10^9/*l*^)	1.4 ± 1.2	1.7 ± 1.3	0.9 ± 0.4^*^
Decreased	34 (29.1%)	13 (16.0%)	21 (58.3%)
Lymphocyte rate (%)	22.9 ± 11.3	27.8 ± 9.9	15.5 ± 7.9^*^
Decreased	45 (38.5%)	17 (19.1%)	28 (77.8%)^*^
CRP (mg/L)	20.1 ± 30.9	10.5 ± 21.3	38.7 ± 36.4^*^
Increased	67 (57.3%)	34 (42.0%)	33 (91.7%)^*^
**CT imaging**			
Interval time from symptoms onset to CT (days)	11 (6–18)	10 (6–17)	13 (9–20)
≥3 Lung lobes affected	82 (70.1%)	48 (59.3%)	34 (94.4%)^*^
> 2 Mixture signs	88 (75.2%)	53 (59.6%)	35 (97.2%)^*^
Total lung severity	4.7 ± 3.6	3.3 ± 2.4	7.8 ± 3.9^*^

* *P < 0.05 vs. common type. Date are n (%), mean ± SD, or median (IQR). CT, computerized tomography; BMI, body mass index; CURB65, confusion, urea, respiratory rate, blood pressure, age 65; CRP, Creative protein*.

### The Scores and Clinical Complications

A total of 18 patients had a MuLBSTA score >12 points and were all of severe type (Table 3). In the severe type patients, 20 (55.6%) were older than 60 years, 14 (38.9%) had hypertension, 7 (19.4%) were smokers, 20 (55.6%) had a lymphocyte count of <0.8*10^9^/L, 30 (83.3%) had multi-lobular infiltrates, and 19 (52.8%) had bacterial coinfection. The frequency of all the terms in severe type patients was higher than that in the common type patients.

**Table 3:** The MuLBSTA score and complications in different type patients.

	**Total**	**Common type**	**Severe type**
	***N* = 117**	***N* = 81**	***N* = 36**
**MuLBSTA score**	8 ± 5	6 ± 4	11 ± 5^*^
**≥12**	18 (15.4%)	0 (0)	18 (50.0%)^*^
Age ≥60 years	35 (29.9%)	15 (18.5%)	20 (55.6%)^*^
Hypertension	19 (16.2%)	5 (6.2%)	14 (38.9%)^*^
Smoker	11 (9.4%)	4 (4.9%)	7 (19.4%)
Lymphocyte <0.8^*^10^9^/L	33 (28.2%)	13 (16.0%)	20 (55.6%)
Multi-lobular infiltrates	66 (56.4%)	36 (44.4%)	30 (83.3%)
Bacterial coinfection	28 (23.9%)	9 (11.1%)	19 (52.8%)^*^
**Complications**			
Electrolyte disturbance	31 (26.5%)	12 (14.8%)	19 (52.8%)^*^
AMI	12 (10.3%)	2 (2.5%)	11 (30.6%)^*^
Respiratory failure	17 (14.5%)	0 (0%)	17 (47.2%)^*^
AKI	3 (2.6%)	1 (1.2%)	2 (5.5%)
ARDS	5 (4.3%)	0 (0%)	5 (13.9%)
Shock	4 (3.4%)	0 (0%)	4 (11.1%)

* *P < 0.05 vs. common type. AMI, acute myocardial injury; AKI, acute kidney injury; ARDS, acute respiratory distress syndrome*.

**Figure 1:**
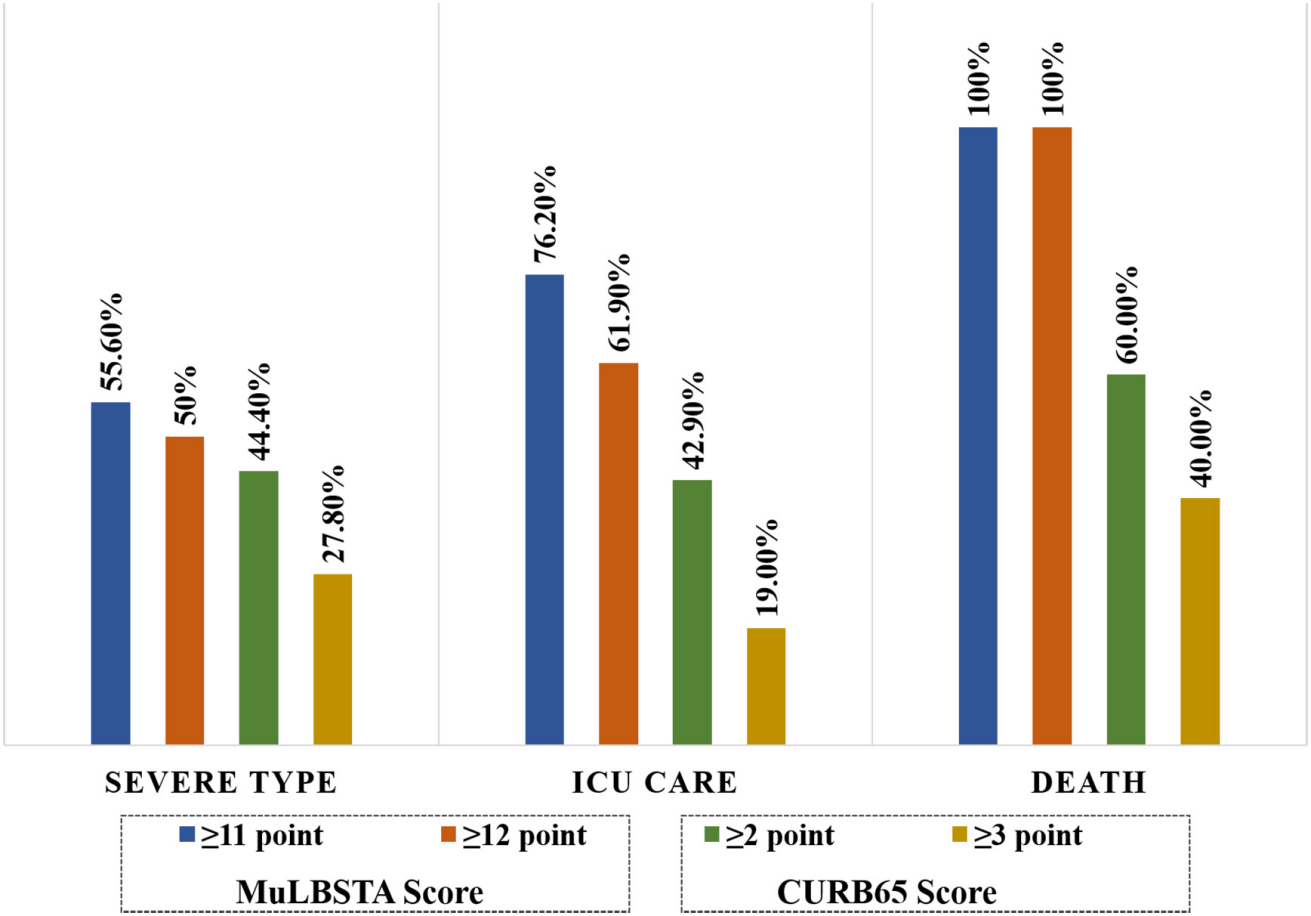
The proportion of different MuLBSTA and CURB65 score points in severe type patients.

For CURB65 score, the mean point in severe type patients were significantly higher than common type patients (*P* < 0.05). Among of 36 severe type patients, there were 16 patients and 10 patients were more than 2 points and 3 points, respectively. In ICU care and death patients, there were 4 (19.0%) patients and 2 (40%) patients had CURB65 score more than 3 points. The proportion of MuLBSTA score more than 12 points was much higher compared with the proportion of CURB65 score more than 3 points in ICU care and deaths (*P* < 0.05) (Figure 1).

During admission, the complications of severe type patients were as follows: 19 (52.8%) patients had with electrolyte disturbance; 11 (30.6%) with AMI; 17 (47.2%) with respiratory failure; 2 (5.5%) with AKI; and 5 (13.9%) with ARDS. Moreover, 4 (11.1%) patients experienced shock and were all of severe type. The frequency of electrolyte disturbance, AMI and respiratory failure in severe type patients were higher than common type patients. The hypokalemia and respiratory failure type I were most common (Table 3).

### Efficacy and Prognosis Value of the MuLBSTA Scale for Death or ICU Care

Of all 21 patients who required ICU care, 13 (61.9%) and 16 (71.9%) had a MuLBSTA score >12 points. The median point of the MuLBSTA score was 13 (IQR, 9, 15). All (100%) patients who died had a MuLBSTA score ≥ 12 points, and the median point was 17 (IQR, 14, 17).

**Figure 2:**
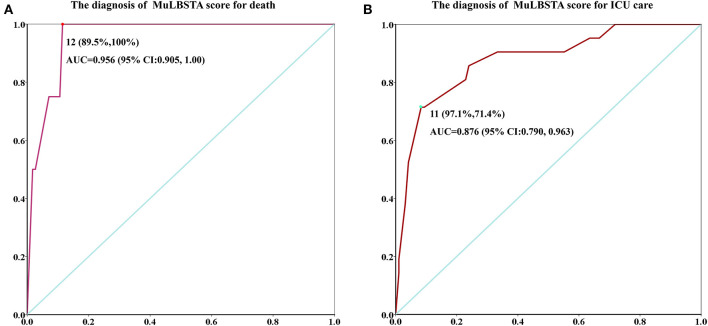
ROC curve of the MuLBSTA score. **(A)** The AUC of death. **(B)** The AUC of ICU.

The diagnosis of the MuLBSTA score for death or ICU treatment is shown in Figure 2. The area under the curve (AUC) of death diagnosis was 0.956, the cutoff value was 12 (specificity, 89.5%; sensitivity, 100%). The AUC of ICU diagnosis was 0.875, and the cutoff value was 11 (specificity, 91.7%; sensitivity, 71.4%).

**Figure 3:**
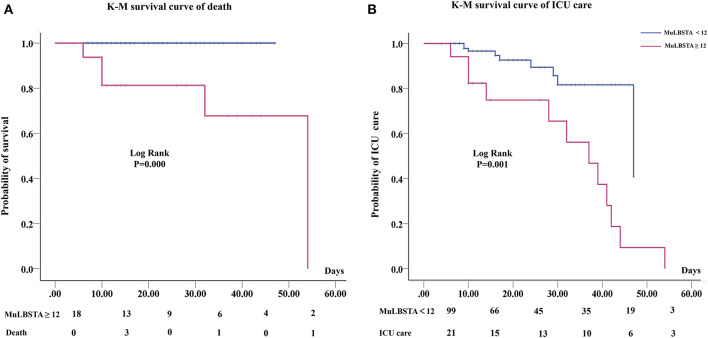
K–M curve of different MuLBSTA scores patients and count of patients in each group at risk at each interval. **(A)** Death risk of MuLBSTA score of ≥12; **(B)** The ICU care needs of MuLBSTA score of ≥12.

The subgroup analysis of the association between the MuLBSTA score and death or ICU care patients were showed in Figure 3. Patients with a MuLBSTA score ≥ 12 had a higher ICU care (log-rank, *P* = 0.001) and higher death (log-rank, *P* = 0.000) risks. The decreasing number of patients at high risk group and the total number of deaths accumulated over time and ICU admissions in the cohort are shown in Figure 3.

## Discussion

In this cohort study, we reported the clinical characteristics and available risk stratification scores associated with the clinical outcomes in patients with COVID-19 pneumonia who died or required ICU care after admission. Patients with a MuLBSTA score ≥12 points were more likely to die or require ICU care. Particularly, severe type patients were more likely to be older, associated with more underlying diseases, severe immune response and lung involvement. These findings suggest that for patients with COVID-19, the MuLBSTA score at first-time hospital admissions may be necessary for risk stratification in patients who have poor prognosis.

As a new type of highly contagious disease in human, this is the first coronavirus to cause a human pandemic (12). The pathophysiology and risk factors of unusually high mortality for COVID-19 have not yet been completely understood. In this study, we validated an effective clinical risk stratification scoring scale-MuLBSTA score for patients infected with SARS-COV-2. This scale is based on the mortality outcomes of 528 patients infected with respiratory viruses according to Guo et al. (9). However, there is no sufficient evidence to verify the efficacy of assessment of poor prognosis in COVID-19 patients (13). The scale is used as an early warning model in predicting mortality in viral pneumonia (9). This scale synthesizes multiple risk factors of the patient, and finally obtains a total score according to the proportions of different risk factor, which is equivalent to the score of the patient's basic condition.

Old age and underlying diseases are now well-known as risk factors in COVID-19 patients, and it has been reported that the SARS-COV-2 infection was more like to occur in older men with comorbidities (13–15). Wu et al. thought that older age was associated with a greater risk of developing ARDS and death, and it may be owing to less rigorous immune response (16). In their cohort, 29.9% of patients were older than 60 years, and 16.2 and 15.4% had associated with hypertension and diabetes. Hypertension and CVD had higher prevalence in the severe cases than in the mild ones. Moreover, no study have demonstrated that a single underlying illness is a risk factor for death or treatment in the ICU at present. Old age or age and underlying disease alone may not be sufficient to determine the risk. In earlier reports increased age in the male population has been associated with higher mortality (17). Smokers are vulnerable to respiratory viruses, and smoking could upregulate angiotensin-converting enzyme 2 receptor levels (17). The prevalence of high smoking level in males may partly explain the higher susceptibility and mortality of male patients.

In addition, virus-induced direct cytopathic effects and viral evasion of host immune responses are believed to play major roles in the severity of coronavirus infection (18, 19). The dysregulation of immune response may result in an excessive inflammation, leading to adverse outcomes (20, 21). Lymphocytopenia was present in 83.2% patients with COVID-19 at admission (22), and severe cases tend to have lower lymphocyte counts (5). In this study, we had similar findings that lymphocyte counts significantly decreased in severe type patients, and more than half of patients had decreased lymphocyte count. Coronaviruses commonly attack the respiratory system and SARS-CoV-2 has been shown to cause lung damage (22, 23). As a significant auxiliary modality, chest CT is a key component of the diagnosis of virus-infected patients (24). It allows the sensitive assessment of lung lesions as well as the degree and location of lung involvement. In previous studies, ground glass and consolidation opacities have been shown to be the most common imaging signs in patients with COVID-19 (8, 23, 25). Although weakened immunity and lung damage are problems in the majority of patients, the effect on death or ICU remains unclear.

The MuLBSTA score is a good diagnostic marker for poor prognosis. In the present study, a score of 12 points indicates the specificity and sensitivity of death were 89.5 and 100%, and 11 points present the specificity and sensitivity of ICU care were 91.7 and 71.4%, respectively. These results strongly suggest that the scale has good efficacy to assess the clinical risk of death and ICU care in patients infected with SARS-COV-2. The survival analysis showed that the higher is the MuLBSTA score, the higher is the death risk. In our results, 50% of severe type patients had a score of ≥12 points, but no common type patients had more than this score. The results implied that severe type patients are more likely to die, and it may be owing to severe immune response and lung involvement.

In clinical practices, there is no effective treatment available for the infected patients, but screening high-risk patients at first admission and appropriate clinical management may be helpful in reducing the incidence of severe complications, such as ARDS or sepsis as well as mortality. Although there are some clinical scales about the severity and risk stratification of pneumonia, such as CURB65 or SOFA score, but in our study, the screening proportion of high-risk patients with MuLBSTA score was higher than that of CURB65 score. Meanwhile, age, hypertension, and smoking status as part of the MuLBSTA score were readily available in the clinical setting, whereas the lymphocyte count and lobe status were assessed by routine blood examination and X-ray or CT scan. Therefore, the score may be a rapid and effective risk stratification strategy.

This study has several limitations. First, the lack of effective antiviral drugs, and all patients underwent different treatment regimens, which may affect the prognosis of patients. Secondly, there may be other risk factors that also affect the prognosis of patients. We verified the validity of the MuLBSTA scale, but the predictive value of a single factor was not analyzed. Finally, because this is the retrospective study, we could only evaluate the short-term prognosis. The long-term prognosis would be analyzed in further studies.

To the best of our knowledge, this is the first retrospective cohort study that focusing on the MuLBSTA score risk stratification of patients with COVID-19 who have experienced a definite outcome. We found that a MuLBSTA score of ≥12 points at admission was a high risk factor for death or ICU care in adult patients with COVID-19. The risk stratification provides the evidence for novel coronavirus clinical interventions in efforts to improve outcomes.

## Conclusions

A higher MuLBSTA score at admission had higher death or ICU risk in patients with COVID-19. The MuLBSTA scale is valuable for the risk stratification of COVID-19 patients, especially regarding death or ICU care.

## Data Availability Statement

All datasets generated for this study are included in the article/supplementary material.

## Ethics Statement

The study was approved by the hospital ethics committee. Written informed consent was waived owing to the rapid emergence of this infectious disease.

## Author Contributions

MY and YG designed the study and takes responsibility for the integrity and accuracy of the data analysis. RX, KH, and KZ contributions to the acquisition, analysis, interpretation of data for the work, and writing of the manuscript. RX, KH, KZ, HX, NZ, HF, LX, RS, LW, HL, and ZY had roles in patient recruitment, data collection, and clinical management. All authors contributed to data acquisition, data analysis, and all reviewed and approved the final version of the manuscript.

## Conflict of Interest

The authors declare that the research was conducted in the absence of any commercial or financial relationships that could be construed as a potential conflict of interest.
